# Fe-Cu Doped Multiwalled Carbon Nanotubes for Fenton-like Degradation of Paracetamol Under Mild Conditions

**DOI:** 10.3390/nano10040749

**Published:** 2020-04-14

**Authors:** Niurka Barrios-Bermúdez, Marta González-Avendaño, Isabel Lado-Touriño, Arisbel Cerpa-Naranjo, María Luisa Rojas-Cervantes

**Affiliations:** 1 Departamento de Química Inorgánica y Química Técnica, Facultad de Ciencias, UNED, Paseo Senda del Rey nº 9, 28040 Madrid, Spain; niurka.barrios@universidadeuropea.es (N.B.-B.); martagonzalezavendano@gmail.com (M.G.-A.); 2 Departamento de Ciencias, Escuela de Ingeniería, Arquitectura y Diseño, Universidad Europea de Madrid, c/ Tajo s/n, Villaviciosa de Odón, 28670 Madrid, Spain; 3 Departamento de Ingeniería Industrial y Aeroespacial, Escuela de Ingeniería, Arquitectura y Diseño, Universidad Europea de Madrid, c/ Tajo s/n, Villaviciosa de Odón, 28670 Madrid, Spain; misabel.lado@universidadeuropea.es (I.L.-T.); arisbel.cerpa@universidadeuropea.es (A.C.-N.)

**Keywords:** Fe-Cu doped carbon nanotubes, paracetamol, Fenton-like reaction

## Abstract

A series of carbon nanotubes doped with Fe and/or Cu, Fe_100−x_Cu_x_/CNT (x = 0, 25, 50, 75 and 100) has been prepared by an easy method of wetness impregnation of commercial multiwalled carbon nanotubes previously oxidized with nitric acid. The wide characterization of the solids by different techniques demonstrates that the incorporation of Fe and Cu to the CNTs has been successfully produced. Fe_100−x_Cu_x_/CNT samples were tested as catalysts in the removal of paracetamol from aqueous solution by a combined process of adsorption and Fenton-like oxidation. Under mild conditions, 25 °C and natural pH of solution, i.e., nearly neutral, values of oxidation of paracetamol between 90.2% and 98.3% were achieved after 5 h of reaction in most of cases. Furthermore, with the samples containing higher amounts of copper, i.e., Cu_100_/CNT and Fe_25_Cu_75_/CNT, only 2 h were necessary to produce depletion values of 73.2% and 87.8%, respectively. The influence of pH and dosage of H_2_O_2_ on the performance has also been studied. A synergic effect between both Cu^+^/Cu^2+^ and Fe^2+^/Fe^3+^ in Fenton-like reaction was observed. These results demonstrate that Fe_100−x_Cu_x_/CNT are powerful Fenton-like catalyst for degradation of paracetamol from aqueous solution and they could be extended to the removal of other organic pollutants.

## 1. Introduction

Advanced oxidation processes (AOPs) are based on the formation of highly reactive radicals capable to degrade recalcitrant organic wastewater contaminants with high efficiency [1,2,3,4]. Among them, heterogeneous processes based on the production of hydroxyl radicals from the decomposition of H_2_O_2_ by the action of catalysts containing the Fe^3+^/Fe^2+^ couple [5,6,7,8,9] or other elements with multiple redox state [10] have been broadly used. They are known as heterogeneous Fenton-like processes and overcome the drawbacks of homogenous counterparts of the limited narrow working pH (3–4) and the necessity of recovering the leached iron from the wastewater. In this sense heterogeneous catalysts containing active species stabilized on oxides [6,11,12,13] zeolites [14,15,16], clays [15,17,18] or carbon materials [19,20,21,22,23] have been widely applied for the degradation of dyes, pesticides, pharmaceuticals and so on. Moreover, magnetite-based catalysts are the most used in the heterogeneous Fenton oxidation, being recently revised [8].

The pharmaceutical compounds are products widely employed throughout the world and their presence in water resources is a worrying environmental issue. Paracetamol (acetaminophen) is one of these pharmaceutical products commonly used for humans as mild analgesics and anti-inflammatories, which is present in waste water treatment plant or even in natural waters [24,25], being necessary to develop efficient treatment processes for reducing its presence in aquatic environments.

Among carbon materials, carbon nanotubes (CNTs) are very interesting as catalysts or alternative supports to the conventional ones due to their excellent properties, such as the high mesoporosity and controlled pore size distribution, hollow and layered structures [26,27]. Furthermore, due to the hydrophobic character of their surface, CNTs exhibit more active sites to interact with organic pollutants. In this regard, different articles have reported the use of iron oxides supported over CNTs as heterogeneous Fenton catalysts for the degradation of phenolic compounds [28,29,30,31], herbicides [32], antibiotics [33] and dyes [34]. However, to the best of our knowledge, there are no studies of Fenton-like degradation of paracetamol using iron catalysts supported on carbon nanotubes. On the other hand, Cu^2+^/Cu^+^ pairs have been proven by other authors [10,35] and by us [13] as efficient catalysts for Fenton-like processes. All above considered, we have thought that the combination of both Fe^3+^/Fe^2+^ and Cu^2+^/Cu^+^ pairs together with the good surface properties of carbon nanotubes could lead to a synergic effect in the performance of this kind of processes. Hence, in this work, we report the facile synthesis of Fe-Cu/CNTs samples prepared by wet impregnation and their use as catalysts in the Fenton-like degradation of paracetamol. The influence of different variables such as the pH of reaction and H_2_O_2_ dosage on the catalytic activity has been investigated. The stability and recyclability of the catalysts, as well as the leaching and mineralization degree have been also studied.

## 2. Materials and Methods 

### 2.1. Preparation of the Fe-Cu Doped Carbon Nanotubes

The commercial pristine multiwalled carbon nanotubes were provided by Sigma-Aldrich (Sigma-Aldrich-Merck, Madrid, Spain) (OD × L 6–9 mm × 5 μm and purity > 95%). They were previously functionalized by oxidation with nitric acid, according to the procedure described in [36]. The commercial carbon nanotubes used as raw material showed CoCu_2_Sn as impurity. However, it was removed after treatment with nitric acid in [36]. The oxidized carbon nanotubes (CNTO) were crushed and sieved to a 0.05 < d < 0.10 mm particle size, and then they were treated with iron and/or copper acetates by the incipient wetness impregnation method. A solution containing the corresponding mix of acetates in the appropriate concentration to obtain a metal loading of 7 wt % (with respect to CNTO) was added drop to drop to 0.40 g of CNTO. The obtained solid was dried at room temperature for 24 h and at 60 °C for 16 h and finally pyrolyzed under nitrogen flow at 400 °C for 30 min. Five catalysts were prepared, with the nominal composition of Fe_100−x_Cu_x_/CNT, with x = 0, 25, 50, 75 and 100.

### 2.2. Characterization of Samples

The textural properties of samples were determined from the nitrogen adsorption–desorption isotherms at −196 °C, by using a Micromeritics ASAP 2010 equipment (Micromeritics, Méringac, France). The samples were previously outgassed at 150 °C for 8 h until a vacuum set point of 200 µm Hg. The surface area and micropore surface were determined by the BET method and *t*-plot method, respectively, and the mesoporosity characteristics of samples were obtained by the BJH method. The morphology of the Fe_1−x_Cu_x_/CNT samples was analyzed by scanning electron microscopy (SEM). Experiments were carried out with a JEOL JSM 6335F microscope (JEOL, Austin, TX, USA) operating at 200 kV. Metal dispersion and nature were followed by high-resolution transmission electron microscopy (HRTEM) using an Oxford Instrument, model: X-Max (Oxford Instruments Nanoanalysis &Asylum Research, High Wycombe, UK) of 80 mm^2^ and resolution between 0.127 and 5.9 KeV and by X-Ray diffraction using a X’Pert Pro Panalytical (Malvern Panalytical, B.V., San Sebastián de los Reyes, Madrid, Spain) diffractometer with CuKα radiation (1.5406 Å), operating at 40 kV and 40 mA.

The content of iron and copper of the samples was determined by inductively coupled plasma optical emission spectrometry (ICP-OES) on an ICP-OES PlasmaQuant^®^ PQ 9000 instrument (Analytic Jena, Upland, CA, USA). The metal leaching after reaction procedure was evaluated by measuring the concentration of metal in the final solution, after filtration through 0.45 mm Durapore membrane syringe filters, by inductively coupled plasma mass spectrometry (ICP-MS) on a Nexion 300D Perkin-Elmer instrument (PerkinElmer INC, Waltham, MA, USA).

### 2.3. Catalytic Activity

The adsorption experiments of paracetamol (PCM) were carried out in a Batch reactor by contacting 10 mg of catalyst with 25 mL of paracetamol solution (50 mg/L, 0.33 mM) under stirring at 700 rpm and 25 °C. The concentration of paracetamol in the solution at selected times was determined by measurements the UV-vis absorption at 243 nm in a Cary-1-UV-VIS (Varian Analytical instruments, Madrid, Spain) spectrophotometer. The adsorbed amount of PCM (C_ads_) was calculated as:

C_ads_ = C_0_ − C_t_ (1)

where C_0_ is the initial concentration of PCM and C_t_ is the concentration of PCM in solution at each selected time, *t*.

The decomposition curves of H_2_O_2_ in absence of paracetamol were determined by contacting 10 mg of catalyst with 25 mL of Milli-Q water and 39.3 μL of H_2_O_2_ (30% Sigma-Aldrich) (Sigma-Aldrich-Merck, Madrid, Spain). Samples were taken periodically and the concentration of H_2_O_2_ was calculated by measuring the absorbance at 405 nm of the yellow complex formed with titanyl sulphate [37]. The amount of decomposed H_2_O_2_ (C_dec_) was calculated as:

C_dec_ = C_t_/C_0_ (2)

where C_0_ is the initial concentration of H_2_O_2_ and C_t_ is the concentration at each selected time, *t*.

In order to carry out the experiments of paracetamol decomposition by H_2_O_2_, 125 mL of paracetamol solution were contacted with 50 mg of catalyst and after adsorption equilibrium (in 30 min) 196 μL of H_2_O_2_ (30%; 13.8 mM) were added. The paracetamol concentration was measured by a high-performance liquid phase chromatograph (HPLC) Agilent Technologies 6120 Quadrupole LC/MS (Agilent Technologies Spain, Las Rozas, Madrid, Spain), equipped with BIN pumps and 6120 Quadrupole LC/MS detection. The separation was achieved on a C18 reverse phase column (Zorbaz RP, Agilent, Agilent Technologies Spain, Las Rozas, Madrid, Spain) using an isocratic mobile phase (50/50 mixture of acetonitrile/water) acidified at pH 2.0 with formic acid, fed at 0.5 mL/min. The amount of decomposed paracetamol was calculated according to Equation (2), by replacing concentrations of H_2_O_2_ by PCM concentrations.

Some experiments of recyclability of catalysts were carried out. After each reaction, the catalysts were filtered off, washed with Milli-Q water and dried at 110 °C in a vacuum oven for 7 h. Due to the loss of catalyst produced between successive cycles, the amounts of H_2_O_2_ and paracetamol were rescaled according to the amount of catalyst.

The pH of the solution was measured at the beginning and at the end of the reaction, but it was not controlled during the same. The amount of H_2_O_2_ used was the corresponding to a concentration of 13.8 mM, twice the stoichiometric one, according to Equation (3):

C_8_H_9_NO_2_ + 21 H_2_O_2_ → 8CO_2_ + HNO_3_ + 25H_2_O (3)

and it was chosen based on previous results [13,21,38]. For a more detailed explanation of the three former procedures see reference [13].

The total organic carbon (TOC) was measured using a Shimadzu TOC-V SCH spectrophotometer (Shimadzu Europa GmbH, Duisburg, Germany). TOC was calculated as the difference between the total carbon (TC) and inorganic carbon (IC) in the liquid samples at selected reaction times.

## 3. Results and Discussion

### 3.1. Characterization of Samples

**Figure 1:**
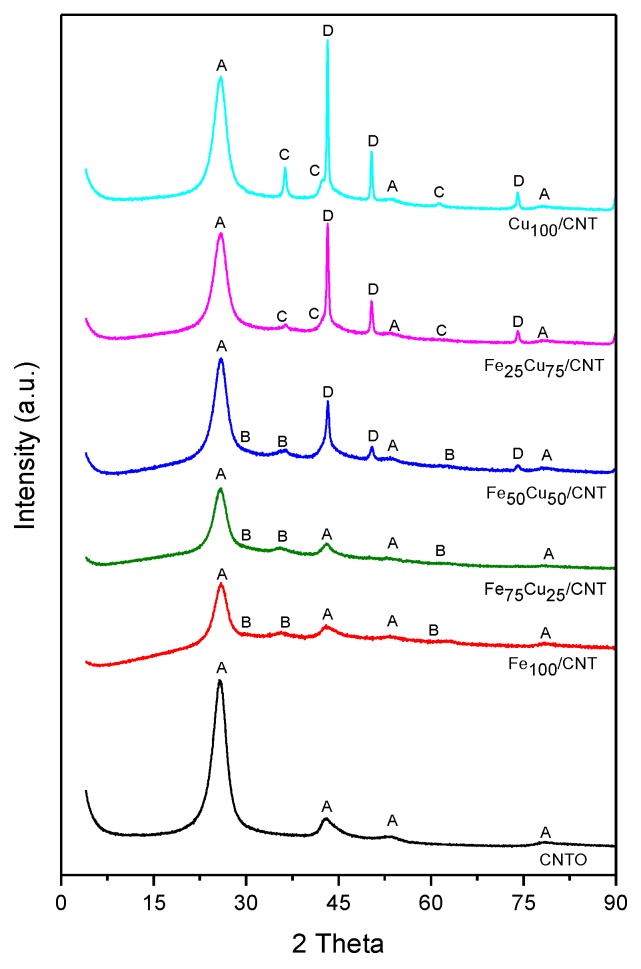
X-ray diffraction patterns of the catalysts. A: graphite; B: Fe_3_O_4_; C: Cu_2_O; D: Cu.

Figure 1 displays the X-Ray diffraction patterns of the catalysts and the crystallite size of the detected phases, calculated by the Debye–Scherrer equation, are given in Table 1. The graphitic nature of the walls of carbon nanotubes was clearly observed by the diffraction peaks detected in all cases at 2θ = 26.2 and 44.4°(JCPDS-ICDD 01-0750-1621), and it was not significantly altered by the incorporation of metals to the nanotubes. For Fe_100−x_Cu_x_/CNT samples, with 100 ≤ x ≤ 50, the diffraction peaks of Fe_3_O_4_ (JCPDS-ICDD 01-079-0418) were observed at 2θ = 30.1, 35.4 and 62.5°. 

The crystallite size of magnetite increases slightly in the order Fe_100_/CNT < Fe_75_Cu_25_/CNT < Fe_50_Cu_50_/CNT, which seem to indicate that the incorporation of increasing amount of copper diminishes the dispersion of Fe_3_O_4_. When the content of one of the metals was 25% with respect to the total metal load, no crystalline phase of that metal species was observed. Thus, any copper phase was detected in Fe_75_Cu_25_/CNT and any iron phase was detected for Fe_25_Cu_75_/CNT. The diffractograms of catalysts containing copper with x ≥ 50 displayed the peaks of metallic copper (JCPDS-ICDD01-085-1326) centered at 2θ = 43.3, 50.4 and 74.1, probably formed due to the reducing character of graphite sheets. The intensity of the main peak of Cu increased when increasing the content of copper in the sample, and the crystallite size also did it, from 18.7 nm for Fe_50_Cu_50_/CNT to 29.0 nm for Cu_100_/CNT. Additionally, Cu_2_O (JCPDS- 03-065-3288) was formed in Fe_25_Cu_75_/CNT and Cu_100_/CNT samples (peaks centered at 2θ = 36.5, 42.3 and 61.5°), with a crystallite size of 15.0 and 20.0 nm, respectively.

From the values of metal content, determined by ICP-OES (Table 2) it can be seen that the total amount was close to the theoretical ones; however, the copper was incorporated in a higher extent than the iron, and their measured values were anomalously higher than the expected. Unfortunately, we did not have an explanation for this fact, except that of a possible error during the preparation of samples.

The textural properties of samples are given in Table 3. Samples are mainly mesoporous, as deduced from the comparison of values of V_mes_ and V_p_ and from the low contribution of S_mic_ to S_BET_ values. The oxidation of carbon nanotubes produces an increment of S_BET_ and pore volume (compare with the values for commercial carbon nanotubes, CNT), mainly of the mesopore volume, because a deagglomeration of nanotubes occurs and a removal of amorphous carbon and impurities from surface is produced. When CNTO are impregnated with the acetates, a decrease in the S_BET_ and V_p_ is produced, due to the blockage of micro- and mesopores by the metallic phases, which have crystallite sizes in this range. However, a clear trend between the content of metal and the S_BET_ values is not observed. As deduced from the shape of the isotherms (see Figure S1) and the average mesopore diameter values, the mesoporous structure of carbon nanotubes seems not to be affected by the impregnation with the metallic salts.

**Figure 2:**
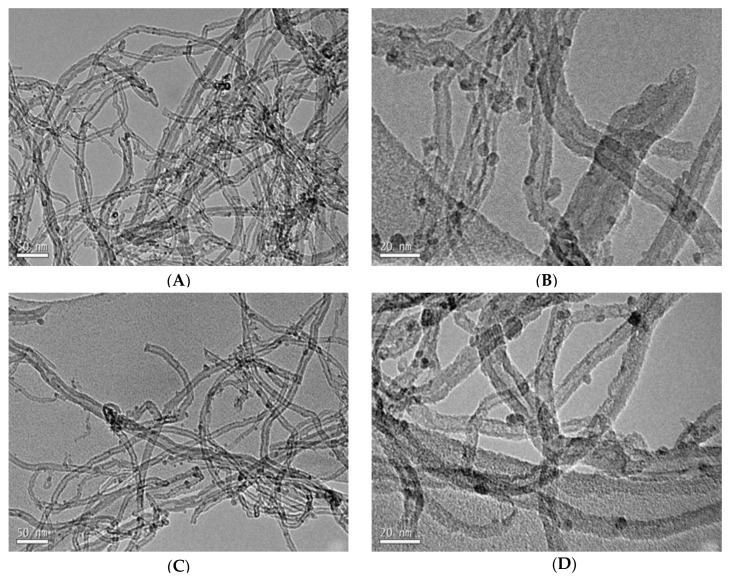

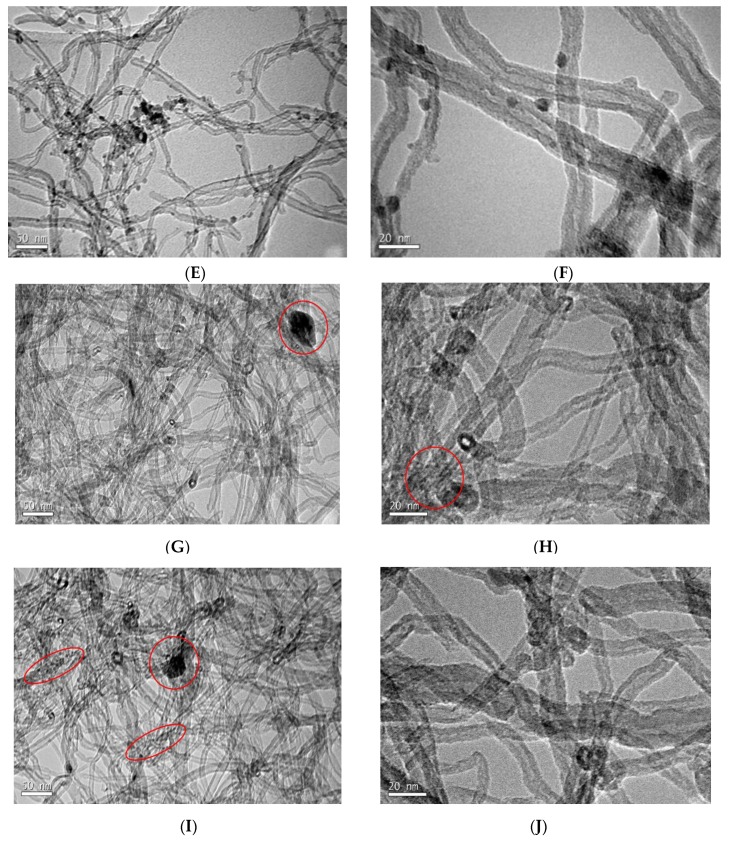
HRTEM images of Fe_100−x_Cu_x_/CNT. (**A**,**B**): Fe_100_/CNT; (**C**,**D**): Fe_75_Cu_25_/CNT; (**E**,**F**): Fe_50_Cu_50_/CNT; (**G**,**H**): Fe_25_Cu_75_/CNT and (**I**,**J**): Cu_100_/CNT. The presence of Cu and/or Cu_2_O particles is marked with red circles in Figures (**G**–**I**).

The morphology and metal dispersion of samples were studied by SEM and HRTEM. The TEM images of catalysts are shown in Figure 2. Fe_3_O_4_ nanoparticles of consistent shape were highly dispersed on the nanotubes in the Fe_100_/CNT catalyst (Figure 2A,B), with a mean particle size (calculated by ImageJ program) of 3.4 nm. These nanoparticles with similar size are also clearly detected in the images of Fe_75_Cu_25_/CNT (4 nm) and Fe_50_Cu_50_/CNT (4.6 nm) samples (Figure 2C–F). The particle sizes observed for Fe_3_O_4_ were very close to those detected by the Scherrer equation (Table 1) and similarly increased slightly in the order Fe_100_/CNT < Fe_75_Cu_25_/CNT < Fe_50_Cu_50_/CNT. However, in the catalysts containing higher amount of copper, i.e., Fe_25_Cu_75_/CNT and Cu_100_/CNT, these smaller particles associated to Fe_3_O_4_ were not observed. For these two catalysts, a combination of very small particles of copper phases (marked with red circles in Figure 2H,I), highly dispersed and hardly detected, together with big particles of aggregates of copper with size around 50–55 nm (also marked with red circles in Figure 2G,I) seems to exist, which indicates a heterogeneous dispersion of particles in these two samples. The incorporation of the metallic phases to the matrix of carbon nanotubes was also corroborated by analyzing the corresponding EDX spectra of TEM images and the EDX spectra of SEM images of the catalysts (Figures S2 and S3, respectively), although the wt % of metal measured was lower than the theoretical one, with the exception of Fe_100_/CNT and Fe_75_Cu_25_/CNT samples, for which it was close to the 7 wt %. The presence of both elements, Fe and Cu, was observed in the EDX spectra of TEM and SEM images (Figures S2 and S3, respectively) of the mixed samples and only one of the elements, Fe or Cu, in the corresponding spectra of monometallic samples. However, in order to know if nanoparticles detected in the spectra of mixed samples are nanocomposites of Fe-Cu, an additional study should be required.

### 3.2. Adsorption of Paracetamol

**Figure 3:**
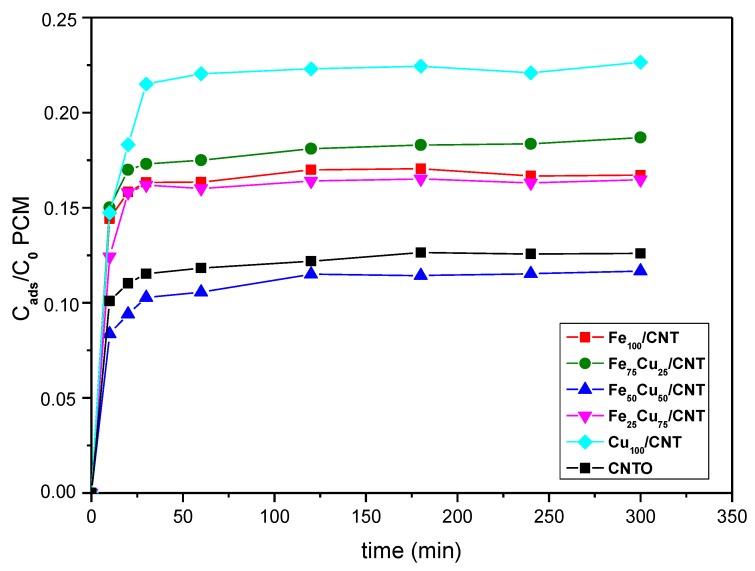
Adsorption kinetics of paracetamol (PCM; C_0_ = 50 mg/L) at 25 °C on CNTO and Fe_100−_Cu_x_/CNT samples.

The kinetics of adsorption of paracetamol for the samples is displayed in Figure 3. The pH of paracetamol solution was about 6.2–6.6, which determines that under these conditions, the paracetamol is in its molecular form. As a result, the adsorption of the organic was produced through weak dispersed forces. Notice that a rapid adsorption occurred in the initial times, in such way that the adsorption equilibrium seemed to be reached at 30 min, and from this time the adsorbed amount kept constant or increased only slightly. The samples that adsorb less paracetamol were those showing the lowest S_mic_ values, that is, Fe_50_Cu_50_/CNT and Fe_25_Cu_75_/CNT. Although the microporosity of these samples was very low or even null, between 11.5% and 14.6% of organic was adsorbed. It should be noticed that the surface of carbon nanotubes had a proportion of basal planes [39] and as a consequence, π–π interactions between carbon nanotubes surfaces and paracetamol aromatic rings must be important in the adsorption process on these materials. For the catalysts containing more micropores, the proportion of basal planes was higher and the π–π interactions would be more relevant. However, the trend observed in the adsorption capacity was not the same that the followed by S_mic_ values; therefore, other factors such as the surface chemistry or the different crystalline phases cannot be discarded in the adsorption process.

As shown in Figure 3, catalysts adsorb between 11.8% and 22.5% of paracetamol at 300 min. However, as said above, the adsorbed amount practically kept constant from 30 min. For that reason, 30 min was selected as the time for equilibrating adsorption process before adding the H_2_O_2_ in the experiments of degradation of paracetamol.

### 3.3. Decomposition of H_2_O_2_

**Figure 4:**
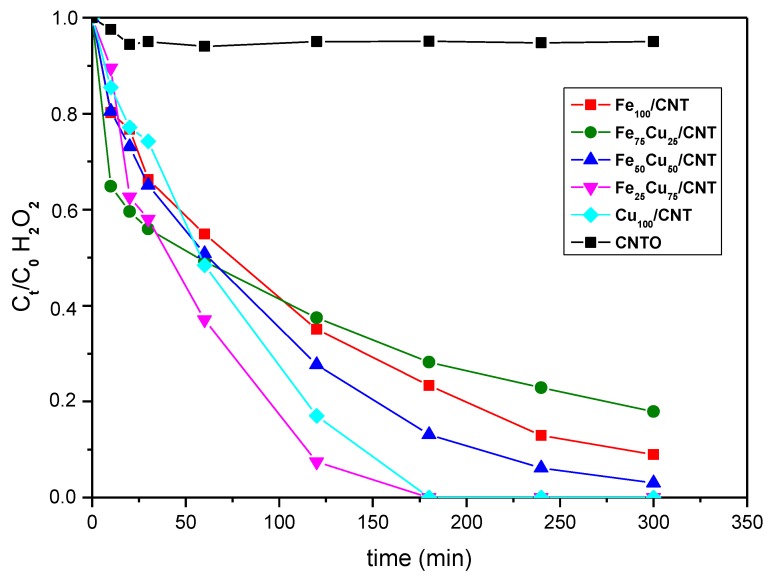
Decomposition kinetics of H_2_O_2_ (C_0_ = 13.8 × 10^−3^ mol/L) in absence of paracetamol at 25 °C on CNTO and Fe_100−x_Cu_x_/CNT samples.

The capacity of the carbon nanotubes for the decomposition of H_2_O_2_ to produce hydroxyl radicals was investigated (Figure 4). It is known that both couples of species, Fe^3+^/Fe^2+^ and Cu^2+^/Cu^+^ are active in heterogeneous Fenton reactions as follows (where S represents the surface of the catalysts) [22,35]:

S-Fe^2+^ + H_2_O_2_ → S-Fe^3+^ + HO^•^ + HO^−^ (4)

S-Fe^3+^ + H_2_O_2_ → S-Fe^2+^+ HO_2_^•^ + H^+^ (5)

S-Fe^2+^ + HO^•^ → S-Fe^3+^ + HO^−^ (6)

S-Fe^3+^ + HO_2_^•^ → S-Fe^2+^ + O_2_ + H^+^ (7)

S-Cu^+^ + H_2_O_2_ → S-Cu^2+^ + HO^•^ + HO^−^ (8)

S-Cu^2+^ + H_2_O_2_ → S-Cu^+^+ HO_2_^•^ + H^+^ (9)

S-Cu^+^ + HO^•^ → S-Cu^2+^ + HO^−^ (10)

S-Cu^2+^ + HO_2_^•^ → S-Cu^+^ + O_2_ + H^+^ (11)

As deduced from Figure 4, the followed order in the decomposition of H_2_O_2_ was Fe_75_Cu_25_/CNT < Fe_100_/CNT < Fe_50_Cu_50_/CNT < Cu_100_/CNT <Fe_25_Cu_75_/CNT. The most active samples were those containing higher amounts of copper, but among them, the best catalyst was that containing also iron, indicating a synergetic effect between the two metals. Copper seems to result in being more active than iron in this reaction. This could be due to the fact that the Cu^+^/Cu^2+^/H_2_O_2_ system can work over a broader pH range, as compared to the Fe^2+^/Fe^3+^/H_2_O_2_ system. Notice that the decomposition of H_2_O_2_ in our work was carried out at the natural pH of paracetamol solution, i.e., near neutral conditions and the Fe^2+^/Fe^3+^ usually works in the 3–4 pH range. In this sense, Cu_100_/CNT decomposed completely the H_2_O_2_ in 180 min, in contrast to the 22% of remaining H_2_O_2_ observed for Fe_100_/CNT at that time. Notice also that CNTO only decomposed a 5% of H_2_O_2_ in all the reaction time, which indicates the activity of the incorporated metals (Fe-Cu) in the Fenton-like reaction under study.

It has been reported that the activity of the catalysts containing iron in the heterogeneous Fenton process depends on characteristics of these oxides, such as crystallinity and surface areas [40,41]. The order observed in the decomposition of H_2_O_2_ for our catalysts was not the followed by the microporosity of samples (see S_mic_ values in Table 3). This means that the catalytic activity was not significantly affected by textural properties and other different factors seem to be involved in it.

As said above, the most active samples were those containing a higher amount of copper, especially Fe_25_Cu_75_/CNT and Cu_100_/CNT, for which Cu_2_O and Cu^0^ were detected by XRD. In the case of Fe_50_Cu_50_/CNT, less active than the former catalysts, Cu_2_O was not detected, but only metallic copper (in addition of Fe_3_O_4_), which suggests that Cu_2_O, with the presence of Cu^+^, which can be oxidized to Cu^2+^ and generate HO^•^ by Equation (8), was more active than Cu^0^ in this reaction. With respect to the catalysts containing iron the presence of the Fe^2+^/Fe^3+^ couple in form of Fe_3_O_4_ was also responsible for the decomposition of H_2_O_2_. Since the rate constant of Equation (8) (k = 1.0 × 10^4^ M^−1^ s^−1^) was much higher than that of Equation (4) (k = 76 M^−1^ s^−1^) [42], samples containing higher amounts of copper showed higher Fenton-like activity than those containing only iron. Furthermore, the synergic effect between Cu and Fe and in general, the better performance of catalysts containing both metals with respect to that of Fe_100_/CNT can also be explained as follows. Fe^3+^ ions were thermodynamically susceptible to be reduced by Cu^+^ ions (Equation (12)). The redox reaction of Cu^+^/Fe^3+^ will accelerate the redox reactions of both Cu^+^/Cu^2+^ and Fe^2+^/Fe^3+^, thus promoting the overall Fenton reaction cycle, and as a result, improving the catalytic rate.

Cu^+^ + Fe^3+^ → Cu^2+^ + Fe^2+^ (12)

This synergic effect was also reported in the degradation of bisphenol A by Wang et al. [22] who found that the catalytic activity of iron-copper bimetallic nanoparticles embedded within ordered mesoporous carbon composite (CuFe-MC) was much higher than that of Fe-MC and Cu-MC.

### 3.4. Fenton-like Decomposition of Paracetamol

**Figure 5:**
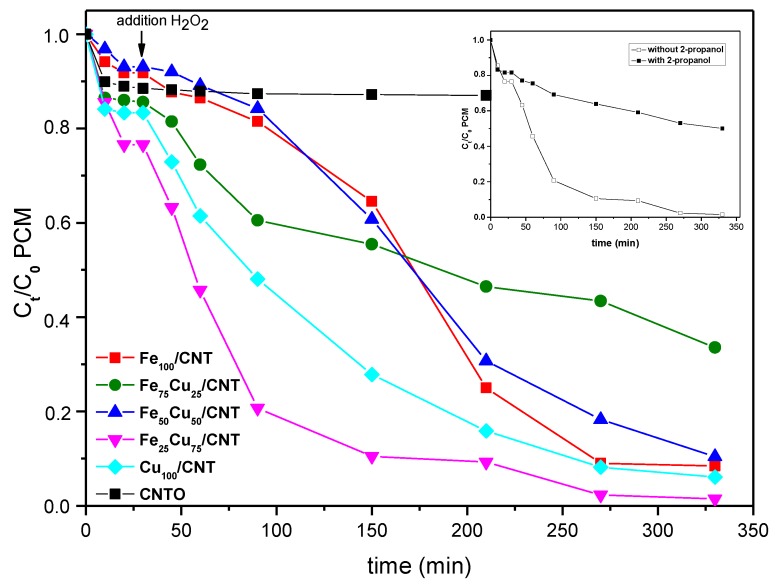
Decomposition kinetics of paracetamol (PCM; C_0_ = 50 mg/L) at 25 °C on Fe_100−x_Cu_x_/CNT samples. C_0_ of H_2_O_2_: 13.8 × 10^−3^ mol/L. Initial pH: 6.3–6.6. Inset: Decomposition kinetics of PCM for Fe_25_Cu_75_/CNT catalyst in the presence and absence of 2-propanol.

The kinetics of catalytic decomposition of paracetamol is depicted in Figure 5. A blank experiment of degradation of paracetamol by H_2_O_2_ in the absence of catalyst was also carried out, and as seen in Figure S4 its degradation was negligible, in this way discarding a direct reaction between both compounds. Two stages were observed in the kinetics of Figure 5. The first one, until 30 min, was due to the physisorption of paracetamol on carbon nanotubes surface, and the values of removed paracetamol (between 7.5% and 23%) were similar to those reported in Figure 3. The second stage produced after the addition of H_2_O_2_ corresponded to the paracetamol decomposition by the Fenton-like process.

**Figure 6:**
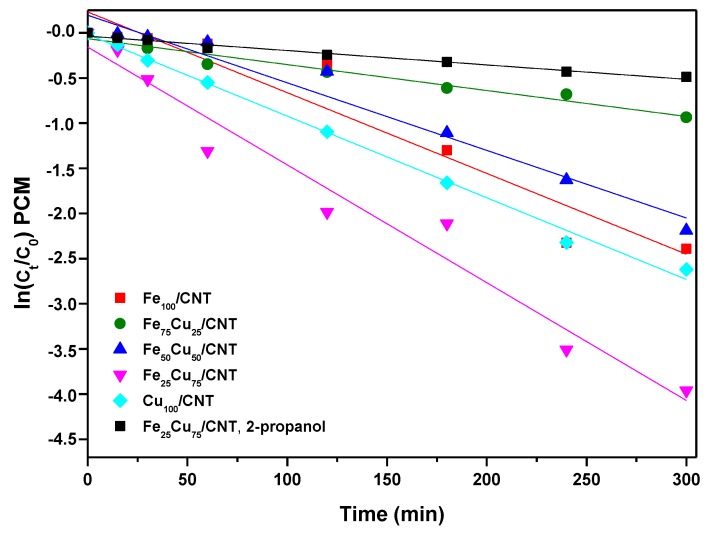
Plots of ln (C_t_/C_0_) versus time for the paracetamol (C_0_ = 50 mg/L) at 25 °C on Fe_100−x_Cu_x_/CNT samples. C_0_ of H_2_O_2_: 13.8 × 10^−3^ mol/L.

As seen in Figure 5, the CNTO did not decompose the PCM and its removal was produced only by adsorption, and a decrease in the paracetamol concentration was not produced after adding H_2_O_2._ The degradation kinetics of paracetamol, considering only the Fenton-like contribution, and not the adsorption step, were adjusted to pseudo-first-order reaction kinetics, according to Equation (13):

ln (C_t_/C_0_) = −k_obs_t (13)

where k_obs_ is the pseudo-first-order apparent rate constant, and the constants were calculated from the slopes of the straight lines by plotting ln (C_t_/C_0_) as a function of removal time (t; Figure 6). As deduced from the values of the constants (Table 4), the order in the kinetics decomposition of paracetamol was Fe_75_Cu_25_/CNT < Fe_50_Cu_50_/CNT < Fe_100_/CNT < Cu_100_/CNT < Fe_25_Cu_75_/CNT. This order was not the same than the followed in the decomposition of H_2_O_2_ in Figure 4. Based on our previous results [13], we thought that the amount of decomposed H_2_O_2_ in the presence of paracetamol could be different than in its absence, and therefore, it would affect to the amount of oxidized paracetamol. In order to check it, during the experiments of degradation of the organic we also registered the decomposition curves of H_2_O_2_, which are represented in Figure S4. By comparison of Figure S4 and Figure 4, it was deduced that in all cases the H_2_O_2_ was decomposed in a less extent in the presence of contaminant than if its absence, and the differences in the rates of decomposition were especially more significant at longer reaction times. It can be explained considering that a competitive effect between paracetamol and H_2_O_2_ by the active species of the catalysts must occur. Similar results were found by us for the decomposition of paracetamol by some perovskites containing copper [13] and by other authors for the decomposition of other organics [35,43].

As deduced from Figure 5, Fe_100−x_Cu_x_/CNT samples were efficient catalysts for the Fenton-like decomposition of paracetamol. Removal of this compound between 90.2% and 98.3% were achieved after 5 h of reaction (330 min including the adsorption step) for all the catalysts, except for Fe_75_Cu_25_/CNT (67% of paracetamol removal). These values were even higher than those obtained previously by us using mesoporous carbons containing iron (78–96%, with a percentage removed by adsorption between 20% and 60%) [21]. Furthermore, for the samples containing higher amounts of copper, that is, Cu_100_/CNT and Fe_25_Cu_75_/CNT, depletion values of 73.2% and 87.8%, respectively, were achieved in only 2 h of reaction.

According to Figure S4, the final amounts of decomposed H_2_O_2_ in presence of paracetamol for F_100_/CNT, Cu_100_/CNT and Fe_25_Cu_75_/CNT samples were very similar (between 75% and 80%) and the amounts or removed paracetamol for these three catalysts (between 92.3% and 98.3%) were also very close between them. However, in the case of Fe_75_Cu_25_/CNT, for which a degradation of paracetamol of 90.3% was achieved, the amount of decomposed H_2_O_2_ (53%) was significantly lower than for the former catalysts. It means that not all the decomposed hydrogen peroxide participated in the oxidation of paracetamol by Equation (14), but some of the generated HO^•^ could react with more H_2_O_2_ by Equation (15), producing an additional decomposition of H_2_O_2_.

Paracetamol + HO^•^ → R^•^ + H_2_O → …→ CO_2_ + H_2_O (14)

H_2_O_2_ + HO^•^ → H_2_O + HO_2_^•^ (15)

It has been proposed that HO**·** radicals are considered as the dominant reactive oxygen species (ROS) in Fenton-like systems. In order to check if these radicals are also the main ROS in our case, a radical scavenger assay was carried out, by testing Fe_25_Cu_75_/CNT in the presence of 2-propanol. As shown in the inset of Figure 5, the removal of paracetamol was strongly inhibited with the presence of the stoichiometric amount of 2-propanol (13.8 × 10^−3^ M) with respect to H_2_O_2_, and the calculated apparent rate constant was significantly reduced from 0.78 to 0.096 h^−1^, suggesting that HO^•^ was the dominant ROS in this study.

#### 3.4.1. Leaching and Mineralization Degree

Determination of leaching of iron and copper was carried out in order to study the possible contribution of homogeneous Fenton to the degradation of paracetamol. The amounts of metals leached off into the aqueous solution after 5 h of reaction, expressed in mg/L, are listed in Table 5. Notice that the concentration of catalyst used in the reaction was 0.4 g/L. When the reaction was carried out at natural pH, for the mixed catalysts, the amount of leached iron, comprised between 0.1 and 0.58 mg/L were below EU guidelines (<2 ppm) [44]. In the case of Fe100/CNT the leaching of iron was higher, 3.99 mg/L; however, it was significantly lower than the values obtained by other authors using Fe_3_O_4_/MWCNT in the degradation of phenol and *p*-nitrophenol (5.25 mg/L) [28] and acid Orange II (29.3% of leached iron) [5]. As a contrast, the amount and percentage of leached copper in the mixed samples were much higher than those values for iron. When comparing catalysts containing only one component, the amount of lixiviated copper in Cu_100_/CNT was lower than that of leached iron in Fe_100_/CNT. The opposite occurs if reaction was carried out at pH 3. The leaching of both iron and copper increased when decreasing pH, but that for copper did it to a greater extent (from 2.17 to 18.61 mg/L), resulting in approximately the double of leached copper with respect to iron.

In order to evaluate the mineralization degree of paracetamol, the TOC values were measured. From the results of Table 6, mineralization degree increased with reaction time; however, the TOC reduction was lower than the corresponding to the paracetamol removal (Figure 5), indicating that this compound was transformed upon oxidation to intermediate products. These products could be some different carboxylic acids, such as ketomalonic, maleic, fumaric, oxalic, oxamic and succinic, which have been reported by different authors [45,46] as products of photo- or electrochemical degradation of the paracetamol. The presence of these acids could explain the decrease in the pH values of solutions along reaction time (Table 5). However, some additional experiments of monitoring of the decomposition products by mass spectrometry should be addressed in future works in order to check this assumption. For some of samples the TOC removal was higher than the obtained (60%) when paracetamol was decomposed by using a zeolite containing iron [47] and those reached (35–50%) in the presence of nanoparticles of iron at pH = 2.6 [38]. Furthermore, in those studies the reaction conditions were much more drastic than that used in our work. Other TOC removal values obtained previously by us for the degradation of paracetamol after 5 h were 52% with a LaCuO_3_ perovskite reference [13] and 45–50% with some Fe-carbon xerogels [13,21,38], which are also lower than the reported in the present work. Therefore, the Fe_x_Cu_100−x_/CNT samples used are efficient catalysts in this Fenton-like process. From the results of Table 6, the best catalyst in terms of both removal and mineralization of paracetamol was Fe_25_Cu_75_/CNT, for which almost all the paracetamol was decomposed and a TOC removal of 85.6% after 300 min was achieved.

#### 3.4.2. Influence of pH and Dosage of H_2_O_2_

**Figure 7:**
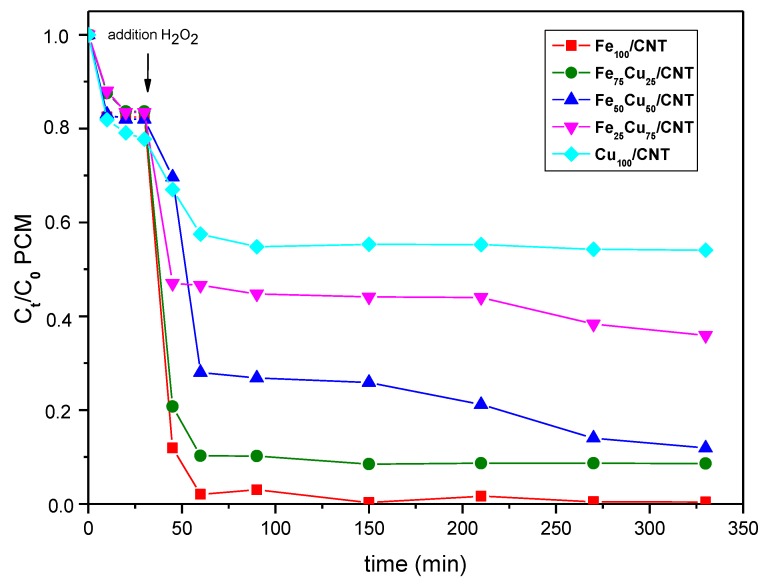
Decomposition kinetics of paracetamol (PCM; C_0_ = 50 mg/L) at 25 °C on Fe_100−x_Cu_x_/CNT samples. C_0_ of H_2_O_2_: 13.8 × 10^−3^ mol/L. Initial pH: 3.

In order to check the influence of the pH of reaction, the initial pH of solution was modified until pH 3 by addition of 10 wt % H_2_SO_4_. The kinetics of the decomposition of paracetamol for all the catalysts under this pH condition is displayed in Figure 7.

Notice that the reaction proceeds better at pH 3 than at natural pH with the catalysts containing higher amounts of iron and faster decompositions of paracetamol were produced (compare Figure 7 and Figure 5); however, the differences in the final decomposition values of paracetamol at both pH were not very significant. On the contrary, the performance of catalysts containing higher amount of copper was worse at pH 3 than at the natural pH of paracetamol solution. In this sense, significantly lower values of decomposition were achieved at acid pH, 64% and 46% for Fe_25_Cu_75_/CNT and Cu_100_/CNT, respectively, in contrast to 87.8% and 73.2% values obtained for those catalysts at natural pH. Furthermore, at pH 3, the catalytic activity decreased with the increment of the amount of copper in the catalysts. As regarding the kinetic curves, two stages could be clearly differentiated, and their corresponding apparent constants, k_1_ and k_2_, are included in Table 4. A very fast decomposition occurred in the first 15–30 min after the addition of H_2_O_2_, and then the reaction was significantly slowed down. From calculated apparent constants (Table 4) it is seen that the first step of the reaction occurred much faster the higher the iron content in samples was.

Additionally, when compared the performance of Cu_100_/CNT and Fe_100_/CNT at pH 3 (Figure 7), the decomposition of paracetamol achieved with the first catalyst was significantly lower (45%) than that for the second (100%), probably due to the higher leaching of copper (18.61 mg/L) with respect to iron (9.25 mg/L; see Table 4), which led to a loss of heterogeneous copper active sites. However, as said above, for Fe_100_/CNT, a faster and higher decomposition of paracetamol occurred at pH 3 with respect to that at natural pH. For this catalyst, the higher amount of leached iron (Table 4) at pH 3 seemed to increase the homogenous contribution to the performance of reaction, also changing the kinetics of reactions, as deduced from the different shape of decomposition curves at the two pH values. Regarding the TOC values (Table S1), in general the final mineralization degrees achieved when the reaction was carried out at pH 3 were lower than the obtained at pH 6.2. Therefore, as a whole, it is better to carry out the reaction at the natural pH, in this way avoiding the additional cost of modifying the pH by adding any acid and decreasing the leached metals.

**Figure 8:**
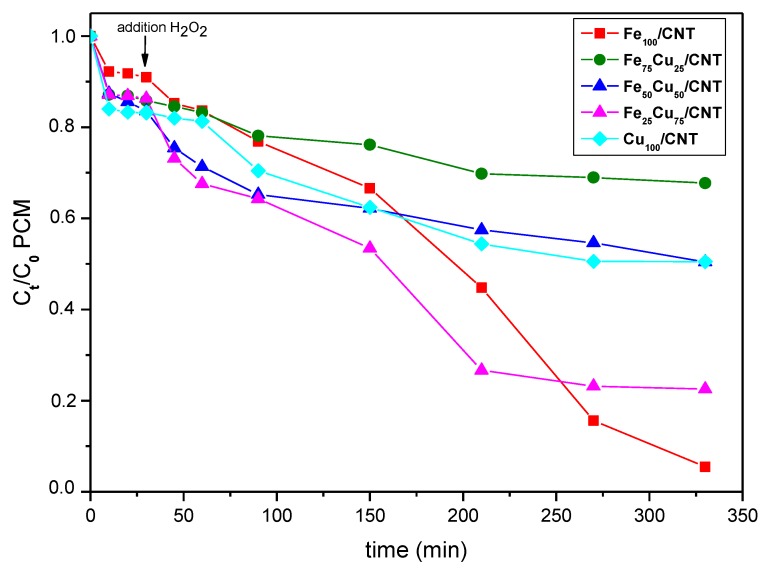
Decomposition kinetics of paracetamol (PCM; C_0_ = 50 mg/L) at 25 °C on Fe_100−x_Cu_x_/CNT samples. C_0_ of H_2_O_2_: 6.9 × 10^−3^ mol/L. Initial pH: 6.3–6.6.

As mentioned in the “Materials and Methods” section, the amount of H_2_O_2_ used in all the experiments was twice the stoichiometric one, according to the previous results. However, we decided to carry out some experiments using the stoichiometric amount, in order to study the influence of the dosage of H_2_O_2_ on the paracetamol decomposition. As deduced from Figure 8, the decomposition values were significantly lower for most of the catalysts when the stoichiometric amount of H_2_O_2_ was used (compare with data from Figure 5) and the apparent constant rate values decreased significantly by a factor of 2.7–4.5 (see Table 4). 

Thus, the paracetamol decomposition was reduced between a 21% (for Fe_25_Cu_75_/CNT) and 52% (for Fe_75_Cu_25_/CNT). The exception was the catalyst containing only iron, i.e., Fe_100_/CNT, for which, the final depletion of paracetamol was almost the same for both H_2_O_2_ dosages, although the decomposition occurred faster when the higher amount of oxidant was used. For this catalyst two stages seemed to exist in the heterogeneous Fenton reaction: the induction period and rapid degradation stage, the degradation rates were accelerated after about 90 min after the adding of H_2_O_2_, as clearly seen by the values of the apparent constant rate calculated for both stages (Table 4).

Furthermore, the values of TOC removal were, in general, lower than those obtained when the double of amount of oxidant was used (see Table S2 in Supplementary Materials). Therefore, as a rule, the use of the stoichiometric amount of H_2_O_2_ decreased significantly the decomposition of paracetamol. Notice that H_2_O_2_ acted as a sink for HO^•^ radicals through Equation (15), decreasing the paracetamol oxidation, and additional H_2_O_2_ amount must be necessary to produce more oxidant radicals through Equations (4), (5), (8) and (9). Consequently, the reaction proceeded better when the H_2_O_2_ amount was the double of the stoichiometric.

#### 3.4.3. Recyclability of Catalysts

**Figure 9:**
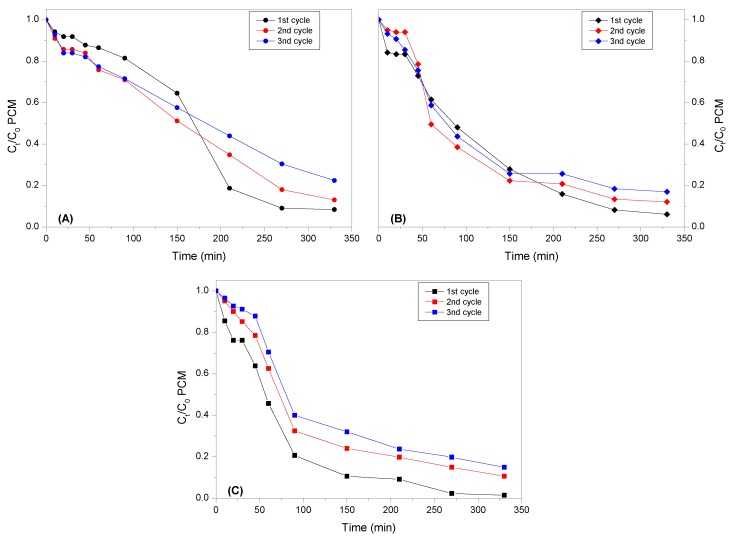
Recyclability of catalysts for the degradation of paracetamol (PCM). (**A**): Fe_100_/CNT; (**B**): Cu_100_/CNT and (**C**): Fe_25_Cu_75_/CNT.

Some experiments for studying the recyclability of the catalysts were carried out, more concretely, with three of the catalysts, Fe_100_/CNT, Cu_100_/CNT and the most active one, Fe_25_Cu_75_/CNT. The results of reusability are shown in Figure 9. 

Notice that a decrease in the activity around 5–6% in the case of Fe_100_/CNT and Cu_100_/CNT, and about 10% for Fe_25_Cu_75_/CNT was produced for the first to the second cycle, probably due to the leaching of metals into solution (Table 4). The reduction of activity after three runs was comprised between 12% (for Cu_100_/CNT) and 15% (for Fe_100_/CNT and Fe_25_Cu_75_/CNT). However, despite the leaching of metals and the decrease in the activity, the TOC removal values were still significant and values between 45% and 78% of mineralization of paracetamol were achieved with recycled catalysts (Table S2). Therefore, the amount of remaining active sites in the catalysts was still enough to carry out the reaction in consecutive cycles.

## 4. Conclusions

In this work, we reported for the first time the preparation of Fe-Cu doped carbon nanotubes by an easy method and their application as efficient catalysts for the degradation of paracetamol (90–98% in 5 h) by a combined process of adsorption and Fenton-like oxidation under mild reaction conditions, 25 °C and pH nearly neutral. The catalysts containing higher amounts of copper, present as Cu_2_O and Cu, showed higher Fenton-like activity than those containing only iron, as Fe_3_O_4_. It could be explained by the higher rate constant of reaction Cu^+^/H_2_O_2_, with respect to that of Fe^2+^/H_2_O_2_, which should produce more HO^•^ oxidant radicals in the case of catalyst with more copper. Additionally, a synergic effect between both Cu^+^/Cu^2+^ and Fe^2+^/Fe^3+^ was produced, and as a result, Fe_25_Cu_75_/CNT was more active than the catalyst with only copper, i.e., Cu_100_/CNT. That catalyst was the best in terms of both removal and mineralization of paracetamol, for which the paracetamol was almost completely decomposed and a TOC removal of 85.6% after 5 h was achieved. The reaction proceeds in all cases better when the H_2_O_2_ amount was double that of the stoichiometric one. The acidification of initial solution until pH 3 exerted a positive effect on the paracetamol degradation when the catalysts containing high amounts of iron were used (probably due to the homogeneous contribution of leached iron) and a negative effect for the samples doped with higher amounts of copper. As a result of the leaching of metals, a decrease of paracetamol degradation around 12–15% occurred from the first to the third reaction cycle. However, the activity and the mineralization degree remained high. These catalysts could be extended to the Fenton-like removal of other contaminants present in waters.

## Figures and Tables

**Table 1:** Crystallite size (nm) of phases detected in oxidized carbon nanotubes (CNTO) and Fe_100−x_Cu_x_/CNT.

Catalyst	Fe_3_O_4_	Cu	Cu_2_O
CNTO	-	-	-
Fe_100_/CNT	2.7	-	-
Fe_75_Cu_25_/CNT	2.9	-	-
Fe_50_Cu_50_/CNT	3.5	18.7	-
Fe_25_Cu_75_/CNT	-	24.9	15.0
Cu_100_/CNT	-	29.0	20.0

**Table 2:** Content of metal (wt %) of Fe_(100−x)_Cu_x_/CNT determined by inductively coupled plasma optical emission spectrometry (ICP-OES) *.

Catalyst	Fe (wt % ± sd)	Cu (wt % ± sd)	(Fe + Cu) (wt %± sd)
Fe_100_/CNT	5.87 ± 0.06 (7)	-	5.87 ± 0.06
Fe_75_Cu_25_/CNT	4.55 ± 0.04 (5.25)	1.93 ± 0.01 (1.75)	6.48 ± 0.04
Fe_50_Cu_50_/CNT	2.91 ± 0.04 (3.5)	3.82 ± 0.01 (3.5)	6.73 ± 0.04
Fe_25_Cu_75_/CNT	1.66 ± 0.01 (1.75)	6.51 ± 0.09 (5.25)	8.17 ± 0.09
Cu_100_/CNT	-	8.07 ± 0.11 (7)	8.07 ± 0.11

* Between brackets: values corresponding to the theoretical ones.

**Table 3:** Textural properties of CNT, CNTO and Fe_100−x_Cu_x_/CNT samples.

Catalyst	S_BET_ (m²/g)	S_mic_ (m²/g)	V_P_ (cm³/g)	V_mes_ (cm³/g)	d_mes_ (nm)
CNT	248.0	24.7	0.799	0.518	12.9
CNTO	329.7	24.2	1.136	0.956	13.8
Fe_100_/CNT	254.6	15.4	0.817	0.707	12.8
Fe_75_Cu_25_/CNT	237.8	4.8	0.775	0.674	13.0
Fe_50_Cu_50_/CNT	273.6	-	0.946	0.839	13.8
Fe_25_Cu_75_/CNT	323.5	0.4	1.073	0.923	13.3
Cu_100_/CNT	306.3	3.3	0.969	0.865	12.7

S_BET_ = specific surface area; S_mic_ = micropore surface area determined by *t*-plot; V_p_ = pore volume at single point at P/P_0_=0.967; V_mes_ = mesopore volume by BJH between 2 and 50 nm; d_mes_ = average mesopore diameter (4V/A) by BJH.

**Table 4:** Values of apparent constants for pseudo-first-order kinetics of paracetamol decomposition. C_0_ of paracetamol: 50 mg/L.

	pH = 6.3–6.6 C_0_ H_2_O_2_ = 13.8 × 10^−3^ mol/L	pH = 3 C_0_ H_2_O_2_ = 13.8 × 10^−3^ mol/L		pH = 6.3–6.6 C_0_ H_2_O_2_ = 6.9 × 10^−3^ mol/L
Catalyst	k (h^−1^)	k_1_ (h^−1^) *	k_2_ (h^−1^) *	k (h^−1^)
Fe_100_/CNT	0.53	7.36	0.34	0.15 (k_1_*), 1.05 (k_2_*)
Fe_75_Cu_25_/CNT	0.17	4.20	0.03	0.05
Fe_50_Cu_50_/CNT	0.45	2.15	0.22	0.10
Fe_25_Cu_75_/CNT	0.78	2.29	0.06	0.29
Cu_100_/CNT	0.54	0.61	0.005	0.12

* Constants for the two stages observed in the kinetic curves.

**Table 5:** Leaching of metals after 5 h of reaction determined by inductively coupled plasma mass spectrometry (ICP-MS).

Catalyst	Leaching of Iron (mg/L)	Leaching of Iron * (%)	Leaching of Copper (mg/L)	Leaching of Copper * (%)
Fe_100_/CNT	3.99	17	-	-
Fe_75_Cu_25_/CNT	0.10	0.5	2.26	29.2
Fe_50_Cu_50_/CNT	0.58	4.95	10.56	69.1
Fe_25_Cu_75_/CNT	0.10	1.4	10.57	40.6
Cu_100_/CNT	-	-	2.17	6.7
Fe_100_/CNT, pH 3	9.25	39.4	-	-
Cu_100_/CNT, pH 3	-	-	18.61	57.6

* Percentage of metal leached off with respect to the initial content in the carbon nanotubes catalysts.

**Table 6:** Values of total organic carbon (TOC) at different reaction times and values of pH of the paracetamol solutions before and after reaction. C_0_ of paracetamol: 50 mg/L; C_0_ of H_2_O_2_: 13.8 × 10^−3^ mol/L.

	TOC (%)				pH	
Catalyst	15 min	60 min	180 min	300 min	Initial	Final
Fe_100_/CNT	-	88.4	70.1	7.1	6.31	5.12
Fe_75_Cu_25_/CNT	-	77.9	54.5	52.5	6.31	4.53
Fe_50_Cu_50_/CNT	79.9	73.7	60.3	59.4	6.31	4.92
Fe_25_Cu_75_/CNT	85.6	43.7	31.3	14.4	6.60	4.35
Cu_100_/CNT	80.7	68.1	62.2	54.0	6.60	4.51

## Supplementary Materials

The following are available online at https://www.mdpi.com/2079-4991/10/4/749/s1, Figure S1: Isotherms of adsorption-desorption of Fe_100−x_Cu_x_/CNT samples. Figure S2: EDX spectra from TEM images of CNTO and Fe_100−x_Cu_x_/CNT samples. Figure S3: SEM images of Fe_100−x_Cu_x_/CNT samples and corresponding EDX spectra. Figure S4: Decomposition kinetics of H_2_O_2_ (C_0_ = 13.8 × 10^−3^ mol/L) in the presence of paracetamol (C_0_ = 50 mg/L) at 25 °C on Fe_100−x_Cu_x_/CNT samples. Table S1: Values of TOC (%) obtained at pH 3 at different reaction times. C_0_ of paracetamol: 50 mg/L; C_0_ H_2_O_2_: 13.8 × 10^−3^ mol/L. Table S2: Values of TOC (%) obtained at natural pH at different reaction times. C_0_ of paracetamol: 50 mg/L.
