# Supplementary material for: Fe-Cu Doped Multiwalled Carbon Nanotubes for Fenton-like Degradation of Paracetamol Under Mild Conditions
Source: Nanomaterials (Basel). 2020 Apr 14;10(4):749. doi: 10.3390/nano10040749 (PMC7221702; doi:10.3390/nano10040749)
Supplement: Supplementary file 1 [file nanomaterials-10-00749-s001.pdf]

nanomaterials

Supplementary Materials

Fe-Cu doped Multiwalled Carbon Nanotubes for Fenton-like Degradation of Paracetamol under Mild Conditions

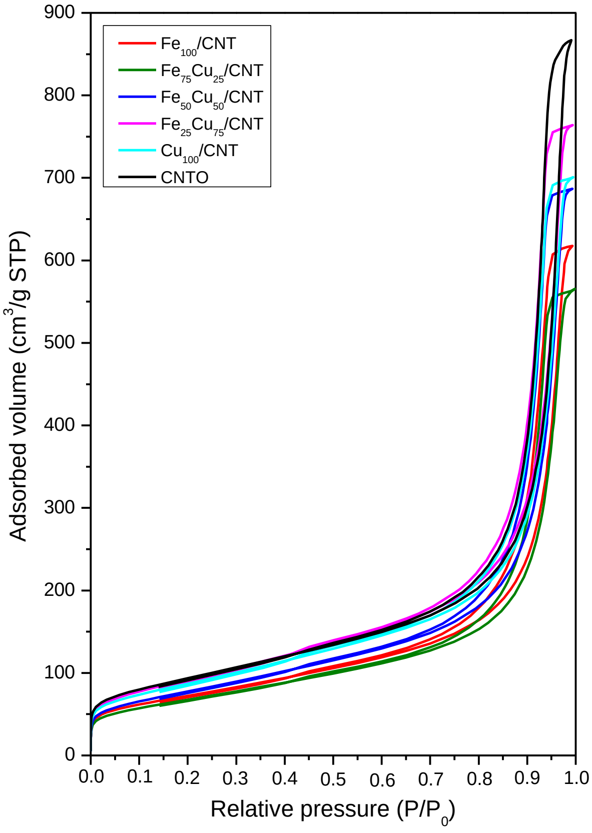

Figure S1. Isotherms of adsorption–desorption of $Fe_{100-x}Cu_x$/CNT samples.

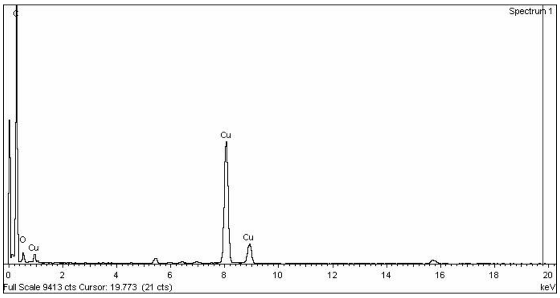

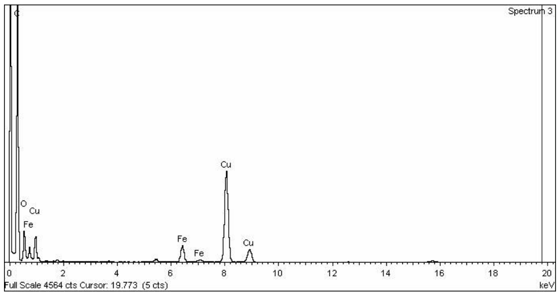

(**A**) (**B**)

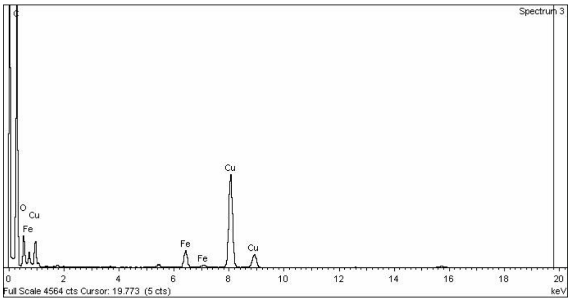

(**C**)

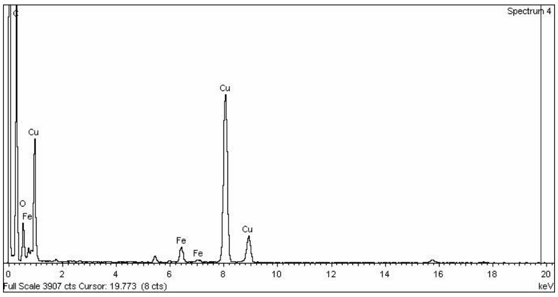

(**D**)

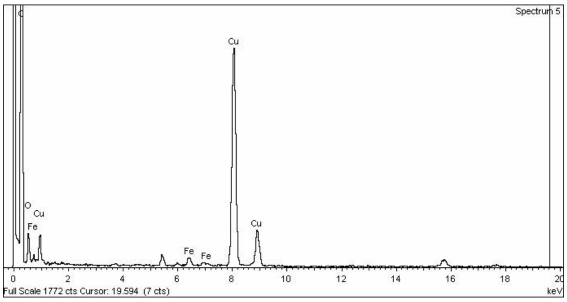

(**E**)

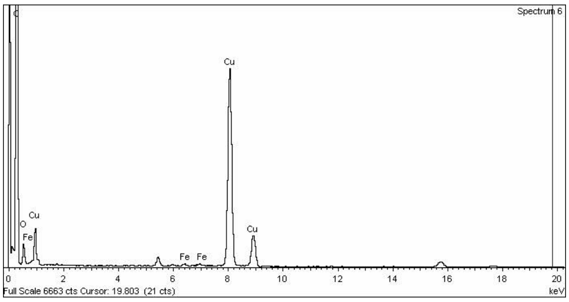

(**F**)

Figure S2. EDX spectra from TEM images of CNTO and $Fe_{100-x}Cu_x$/CNT samples. (**A**): CNTO; (**B**): Fe_{100}/CNT; (**C**): $Fe_{75}Cu_{25}$/CNT; (**D**) $Fe_{50}Cu_{50}$/CNT; (**E**) $Fe_{25}Cu_{75}$/CNT and (**F**) Cu_{100}/CNT. In order to consider the contribution of copper grid, the EDX spectrum of CNTO, which does not contain copper, is included.

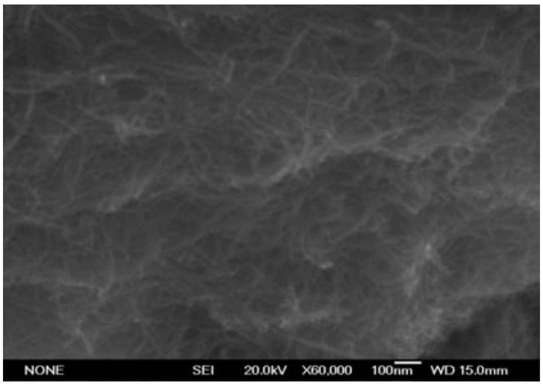

(**A**)

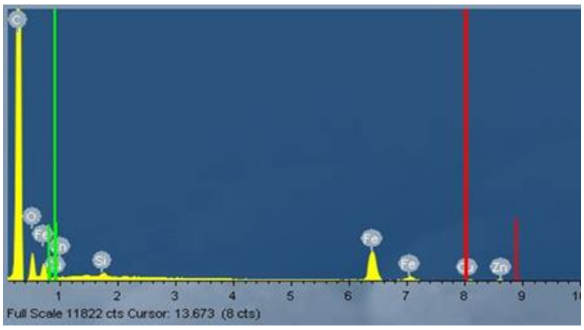

Weight %: C: 80.56; O: 11.99; Fe: 7.45.

(**B**)

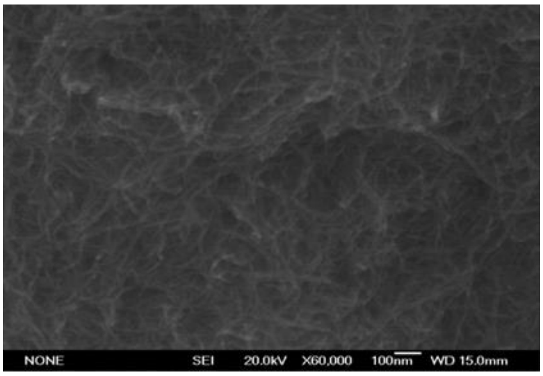

(**C**)

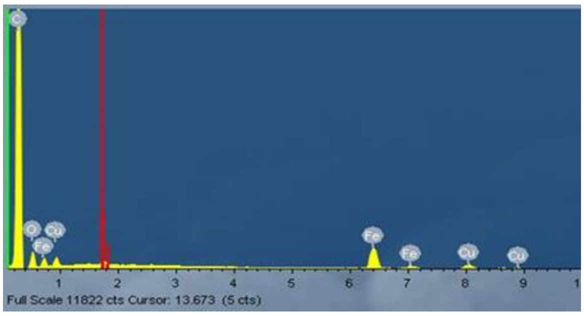

Weight %: C: 82.65; O: 9.12; Fe: 5.95; Cu: 2.28

(**D**)

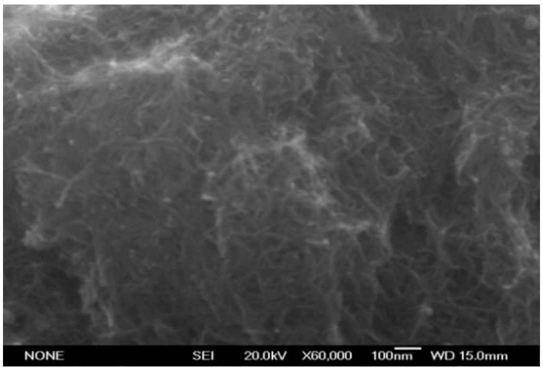

(**E**)

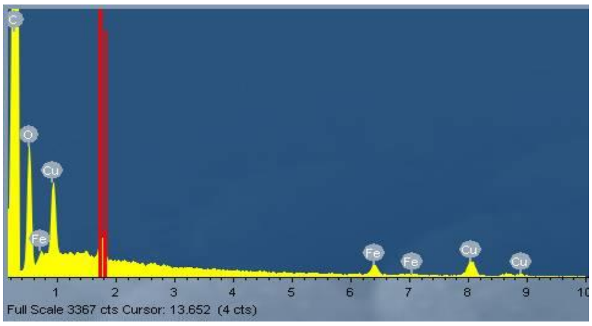

Weight %: C: 88.65; O: 9.87; Fe: 0.36; Cu: 1.12

(**F**)

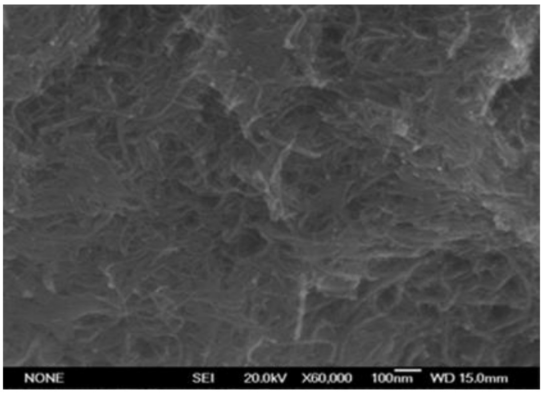

(**G**)

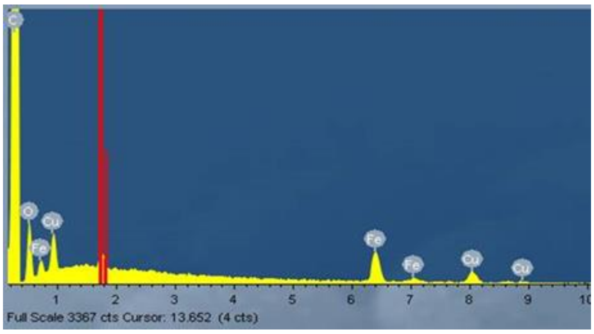

Weight %: C: 88.5; O: 8.04; Fe: 2.01; Cu: 1.45.

(**H**)

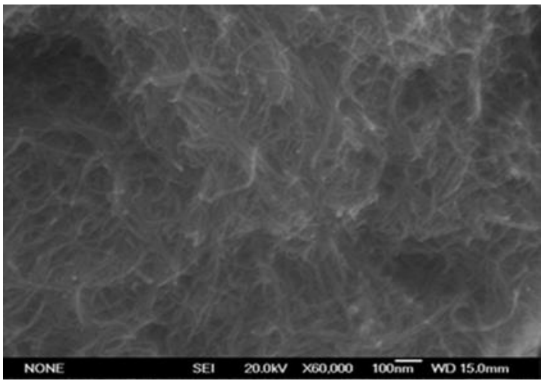

(**I**)

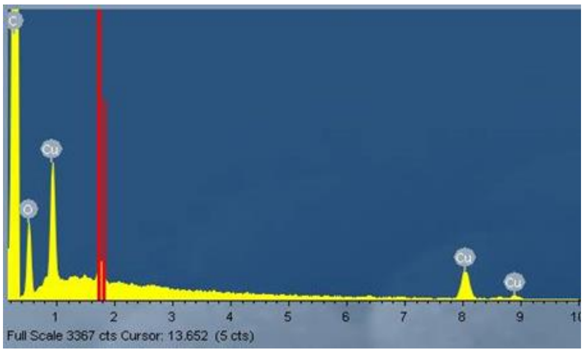

Weight %: C: 90.28; O: 7.08; Cu: 2.64

(**J**)

Figure S3. SEM images of $Fe_{100-x}Cu_x$/CNT samples and corresponding EDX spectra. (**A**,**B**): Fe_{100}/CNT; (**C**,**D**): $Fe_{75}Cu_{25}$/CNT; (**E**,**F**): $Fe_{50}Cu_{50}$/CNT; (**G**,**H**): $Fe_{25}Cu_{75}$/CNT and (**I**,**J**) Cu_{100}/CNT.

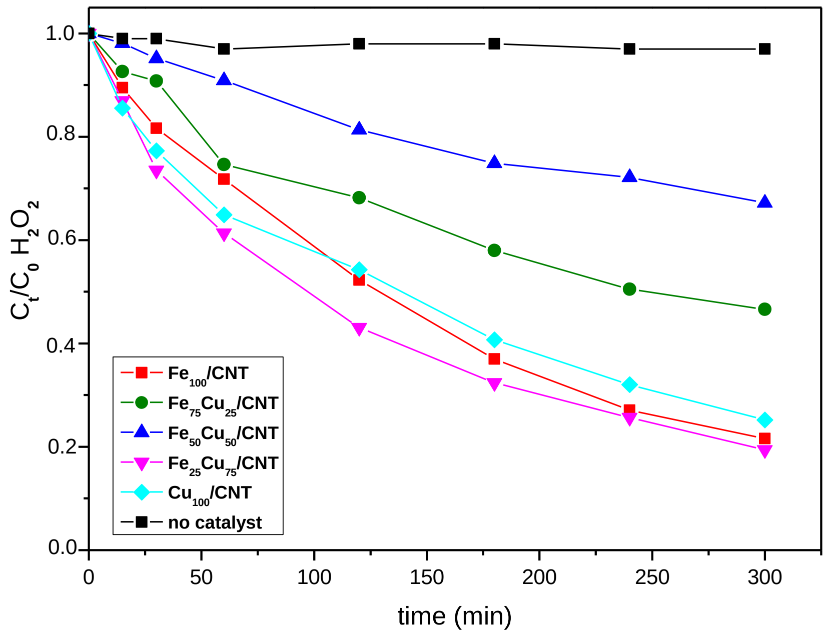

Figure S4. Decomposition kinetics of H_2O_2 (C_0 = 13.8 × 10^{-3} mol/L) in the presence of paracetamol (C_0 = 50 mg/L) at 25 °C on $Fe_{100-x}Cu_x$/CNT samples.

Table S1. Values of TOC (%) obtained at pH 3 at different reaction times. C_0 of paracetamol: 50 mg/L; C_0 H_2O_2: 13.8 × 10^{-3} mol/L.

Catalyst	15 min	60 min	180 min	300 min
Fe_{100}/CNT	68.7	62.6	59.8	55.2
$Fe_{75}Cu_{25}$/CNT	64.5	64.3	63.4	58.7
$Fe_{50}Cu_{100}$/CNT	70.9	67.3	59.6	53.7
$Fe_{25}Cu_{75}$/CNT	83.7	62.7	46.4	26.4
Cu_{100}/CNT	86.6	84.4	78.1	73.2

Table S2. Values of TOC (%) obtained at natural pH at different reaction times. C_0 of paracetamol: 50 mg/L.

Catalyst	15 min	60 min	180 min	300 min
Fe_{100}/CNT (C_0 H_2O_2: 6.9 × 10^{-3} mol/L)	88.3	86.6	76.5	59.6
$Fe_{75}Cu_{25}$/CNT (C_0 H_2O_2: 6.9 × 10^{-3} mol/L)	96.4	94.4	86.7	81.7
$Fe_{50}Cu_{50}$/CNT (C_0 H_2O_2: 6.9 × 10^{-3} mol/L)	72.3	70.1	66.6	65.9
$Fe_{25}Cu_{75}$/CNT (C_0 H_2O_2: 6.9 × 10^{-3} mol/L)	84.0	73.3	70.8	20.3
Cu_{100}/CNT (C_0 H_2O_2: 6.9 × 10^{-3} mol/L)	85.0	82.4	67.1	55.0
Fe_{100}/CNT, 2rd run (C_0 H_2O_2: 13.8 × 10^{-3} mol/L)	92.3	87.5	72.4	54.6
$Fe_{25}Cu_{75}$/CNT, 2rd run (C_0 H_2O_2: 13.8 × 10^{-3} mol/L)	87.1	62.0	43.2	22.0
Cu_{100}/CNT, 2rd run (C_0 H_2O_2: 13.8 × 10^{-3} mol/L)	93.7	80.7	63.1	50.2
